# Measuring cognitive flexibility in anorexia nervosa: Wisconsin Card Sorting Test versus cued task-switching

**DOI:** 10.1007/s40519-023-01589-6

**Published:** 2023-07-18

**Authors:** Kelly M. Dann, Aaron Veldre, Stephanie Miles, Philip Sumner, Phillipa Hay, Stephen Touyz

**Affiliations:** 1 School of Psychology, The University of Sydney, Brennan MacCallum Building (A18), Sydney, NSW 2006 Australia; 2 Faculty of Medicine and Health, InsideOut Institute for Eating Disorders, The University of Sydney and Sydney Local Area Health District, Sydney, Australia; 3 School of Psychological Sciences, Macquarie University, Sydney, Australia; 4 Centre for Mental Health, Swinburne University of Technology, Melbourne, Australia; 5 Orygen, Melbourne, Australia; 6 Centre for Youth Mental Health, The University of Melbourne, Melbourne, Australia; 7 Translational Health Research Institute, School of Medicine, Western Sydney University and Mental Health Services SWSLHD, Campbelltown, Australia

**Keywords:** Anorexia nervosa, Eating disorders, Cognition, Cognitive flexibility, Task-switching

## Abstract

**Purpose:**

The Wisconsin Card Sorting Test (WCST) is the most common measure of cognitive flexibility in anorexia nervosa (AN), but task-switching paradigms are beginning to be utilized. The current study directly compared performance on a cued task-switching measure and the WCST to evaluate their association in participants with a lifetime diagnosis of AN, and to assess which measure is more strongly associated with clinical symptoms.

**Methods:**

Forty-five women with a lifetime diagnosis of AN completed the WCST, cued color-shape task-switching paradigm, Anti-saccade Keyboard Task, Running Memory Span, Eating Disorder Examination Questionnaire, Depression Anxiety Stress Scales short form and Eating Disorder Flexibility Index.

**Results:**

There was no evidence of a significant association between WCST perseverative errors and cued task-switching switch costs. Results suggest lower working memory capacity is a determinant of higher perseverative error rate. When controlling for mood variables, neither cognitive flexibility measure was a significant independent predictor of symptom severity.

**Conclusions:**

Results provide support for previous suggestions that WCST perseverative errors could occur due to difficulties with working memory, sensitivity to feedback, and issues with concept formation. Cued task-switching paradigms may provide a useful measure of cognitive flexibility for future eating disorders research by reducing task-specific confounds.

**Level of evidence:**

Level III Case–control analytic study.

**Supplementary Information:**

The online version contains supplementary material available at 10.1007/s40519-023-01589-6.

## Introduction

Cognitive flexibility is a component of executive function, the set of higher order cognitive processes that control goal-related thought and action [8]. Tests of cognitive flexibility assess the ability to flexibly switch between mental task sets according to changes in current goals. Individuals with anorexia nervosa (AN) perform more poorly than control groups on a number of cognitive flexibility tasks [43]. Lower cognitive flexibility scores versus matched controls have also been found for participants with a past diagnosis of AN [22], across subtypes of AN [34], and in unaffected sisters and unaffected mothers of individuals with AN [15, 18]. Cognitive inflexibility is, therefore, theorized to be a risk factor associated with the development and maintenance of the disorder (e.g., Cognitive–Interpersonal Maintenance Model of AN) [31, 39].

**Fig. 1:**
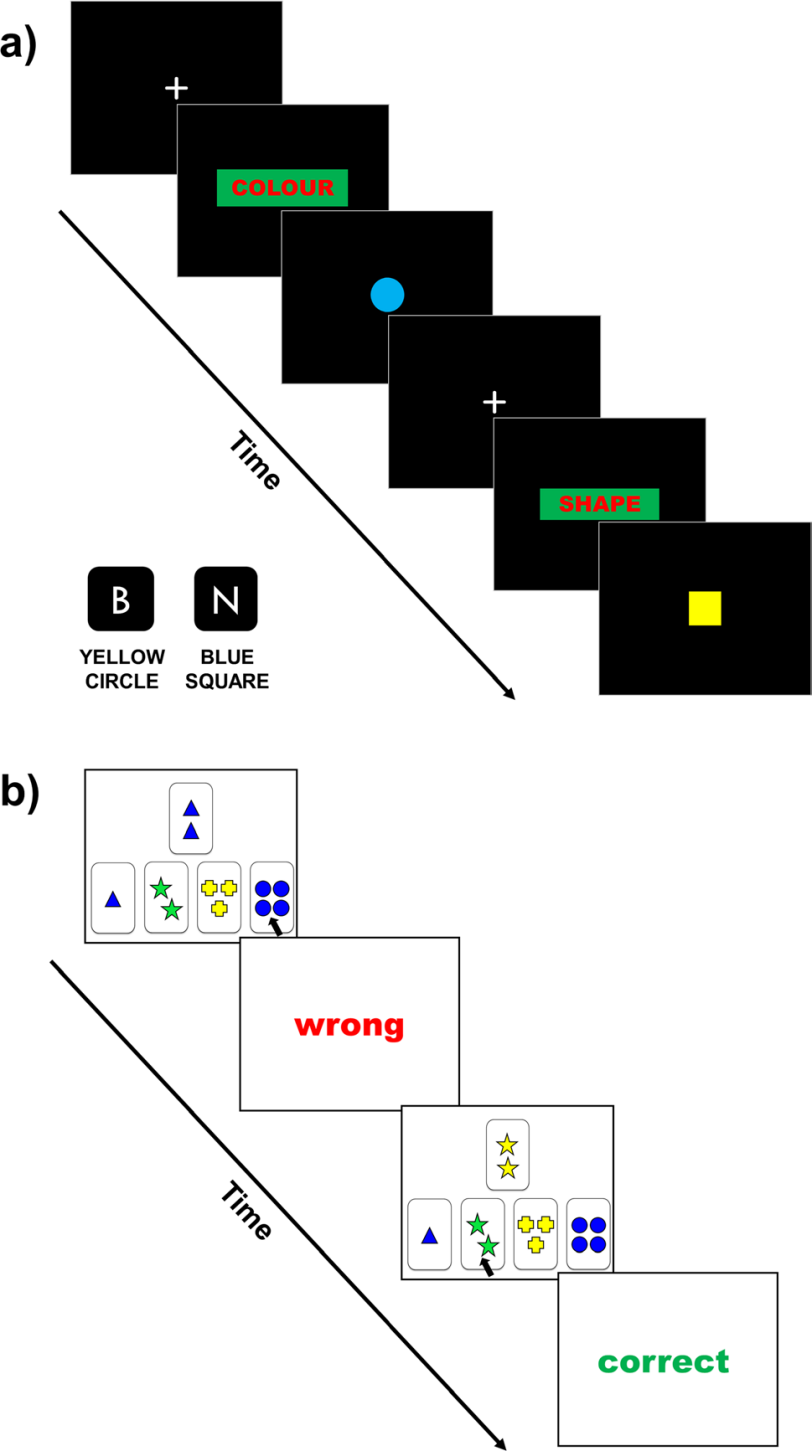
Schematic diagram of a typical trial sequence in both the colour-shape task-switching paradigm and the Wisconsin Card Sorting Test. **a** Colour-Shape Task-Switching Paradigm example trial sequence including a task-switch: fixation cross; task cue; bivalent stimulus; participant responds using the B and N key on a standard keypad to indicate their response. **b** Wisconsin Card Sorting Test example trial sequence: participant sorts response card Blue Triangle Two by colour using mouse click; receives feedback that this is the wrong rule; participant sorts response card Yellow Star Two by shape; receives feedback that this is correct. Stimuli are not to scale

The Wisconsin Card Sorting Test (WCST) [12], a widely used neuropsychological test of executive function, is the most commonly administered measure of cognitive flexibility in AN [22]. In the WCST, participants are required to sort a response card by color, shape, or number according to a rule that is learned through trial, error, and feedback. After several cards, the correct sorting dimension changes without warning, requiring a flexible shift to a new rule (see Fig. 1). Cognitive flexibility deficits are indexed by *perseverative errors*, i.e., errors in sorting after the participant has been given enough information to derive the correct rule. Meta-analytic results suggest that participants with AN make more perseverative errors in the WCST than matched controls with medium effect (Hedge’s *g* = − 0.42) [35]. Although the WCST has clinical utility as a test of executive function, it has limited utility in isolating specific cognitive flexibility impairments versus issues with associated components of cognitive control, such as updating working memory or inhibition of a response [25], or from difficulties with reversal learning [42].

Task-switching paradigms were specifically developed to parse the multiple cognitive control processes involved in switching between task sets, and are the most widely used measure of cognitive flexibility in the general population experimental literature (see [21, 28] for reviews). In a typical cued task-switching paradigm, the participant is presented with a bivalent stimulus (e.g., a red circle) to which they must perform one of two tasks—either respond to indicate the color or respond to indicate the shape. Sequences of trials include runs, where the task stays the same from one trial to the next (*repeat trials*, e.g., color, color) and where the task changes from the previous trial (*switch trials*; e.g., color, shape, see Fig. 1). Responding is typically slower on switch trials than repeat trials, and this *switch cost* is used as a measure of the flexibility of the cognitive control system in updating goal-related responses based on changing demands.

Although most cognitive flexibility data in AN is from the WCST, task-switching paradigms are beginning to be utilized. Berner et al. [2] observed higher switch costs, indicating less flexibility, in participants with current AN compared to controls. Other studies found no differences in switch costs, but reported differences in response speed, error rates, and patterns of brain activity associated with switching between tasks [17, 40, 41]. However, because the WCST and task-switching have different outcome variables, it is unclear how to reconcile task-switching data with the existing literature.

Consistent with the assumption that both tasks measure cognitive flexibility, switch costs have been found to predict WCST perseverative errors in the general population—both in young adults [25, 26], and older adults [11]—over and above the variance accounted for by measures of working memory and inhibitory control. Although the relationship between switch costs and WCST perseverative errors has not been explicitly evaluated in AN, Van Autreve et al. [40] reported non-significant associations between the measures in participants with AN. Whether task-switching and the WCST can be used interchangeably as measures of cognitive flexibility for future research in AN, therefore, requires further systematic investigation.

## Aim and hypotheses

The current study aimed to (1) directly compare performance on a cued task-switching measure and the WCST to evaluate their association in participants with a lifetime diagnosis of AN, and (2) assess which measure is more strongly associated with clinical symptoms. Consistent with findings in the general population [11, 25, 26], we hypothesized that switch costs would be a significant independent predictor of perseverative error scores. Because all behavioral tasks measure associated cognitive and task-related processes to some extent (i.e., the *task impurity problem*), we also assessed the relative contribution of inhibitory control and working memory capacity to scores, consistent with standard practice in general population research, and asked participants to report on their experience with the task. Following previous recommendations for cognitive flexibility research in AN [1], we concurrently assessed depression, anxiety, and stress. To further assess the relative utility of these tests for the AN population, we explored associations with self-reported flexibility in everyday life.

## Methods

### Participants and procedure

Forty-five women with a lifetime diagnosis of AN were recruited from the community between April 2021 and May 2022 through advertisements on eating disorder organization websites. Recruitment was open to all genders but only women responded. To be eligible for the study, participants were required to be 18 years or older and have received a formal diagnosis of AN from a medical professional. Participants with a partial AN syndrome diagnosis (OSFED-AN, Atypical AN) were included. Diagnostic information was collected via self-report. Participants provided informed consent before completing the study, which was administered online with telephone proctoring throughout a 1-h testing session to ensure participants were set up correctly, followed the task order, and had the same opportunity to ask questions as is standard in laboratory-based testing. Task order was fixed to minimize any measurement error due to Participant × Order interactions [26]. This research was conducted in line with the principles of the Declaration of Helsinki and with approval from The University of Sydney Human Research Ethics Committee (2021/020).

#### Power analysis

An a priori power analysis conducted using G-Power [10] based on the effect size for a comparison of WCST scores and switch costs [11] indicated our study required a sample size of 43 participants given an alpha of 0.05 at 80% power.

### Measures

***Wisconsin Card Sorting Test*** [13] measured cognitive flexibility and was administered via Inquisit 6 [computer software] (2021) [24]. Participants sort a response card to match one of four stimulus cards that vary on three dimensions (color, shape, number). The sorting dimension changed after 10 correct responses, and the task ended when participants correctly sorted by the three dimensions twice, or when all 128 target cards had been presented. The dependent variable was percentage of perseverative errors, scored according to Heaton et al. [13]. Following the task, participants were asked to report whether they had ever done the task before, whether they found it easy or difficult, and why.

***Cued color-shape task-switching paradigm*** [20] measured cognitive flexibility and was administered via PsyToolkit [36, 37]. Participants were presented with a bivalent stimulus (e.g., a yellow circle) to which they applied one of two cued tasks (respond to the color; respond to the shape). Participants responded with two keys on a standard keypad, where each key indicated a color (yellow or blue) and a shape (circle or square). Participants completed 10 practice trials followed by 200 test trials. Error trials, post-error trials, and trials with RTs above 2000 ms were excluded from the analysis. The dependent variable was switch cost (mean RT for repeat trials—mean RT for switch trials).

***Anti-saccade keyboard task*** [32] measured inhibitory control and was administered via Inquisit 6 [computer software] (2021) [24]. Participants were instructed to not look towards an abrupt-onset cue and instead look to the opposite side of the screen, where a target stimulus (a left-, right- or up-pointing arrow) was presented for 175 ms. Participants indicated the direction of the arrow using a standard keypad for 18 practice trials followed by 90 test trials. Higher error rate indicates poorer inhibitory control.

***Running memory span task*** [3] measured working memory capacity and was administered via Inquisit 6 [computer software] (2021) [24]. Participants were presented with a series of letters that varied in run length (3–8) and were cued to recall between 3 and 6 of the last letters in the sequence in the order that they were presented. Higher span scores indicate greater working memory capacity.

***The Eating Disorder Examination Questionnaire*** (EDE-Q) [9] assessed eating disorder attitudes and behaviors over the past 28 days. Higher scores indicate more severe eating disorder pathology. Height and weight were reported, and BMI (kg/m^2^) was calculated from this data. Cronbach’s alpha of 0.93 has been reported in an Australian female community sample [27], and in this study was 0.95.

***The Eating Disorder Flexibility Index*** (EDFLIX) [5] assessed general and eating disorder-specific cognitive–behavioral flexibility in everyday life. Total scores range from 36 to 216, with higher scores indicating greater flexibility. Cronbach’s alpha for a mixed ED sample reported in the validation study was 0.91 [5], and in this study was 0.95.

***The Depression Anxiety Stress Scales short form*** (DASS-21) [19] assessed mood symptoms over the past week. Higher scores indicate greater psychopathology. Cronbach’s alpha of the depression, anxiety, and stress scales in a general population sample have been reported as 0.88, 0.82, and 0.90, respectively [14], and in this study were 0.94, 0.81, 0.87.

#### Data analysis

Data analysis was conducted using IBM SPSS Statistics 28.0 software. The analytic plan was preregistered at Open Science Framework (https://osf.io/nbvrd). Performance data were inspected for normality and outliers (> 3 *SD*) and found to be within acceptable parameters. Linear multiple regression was conducted to test whether WCST scores were predicted by switch costs on the task-switching measure while controlling for associated executive functions; inhibitory control (anti-saccade errors) and working memory capacity (span scores). Linear multiple regression was conducted to test whether EDE-Q scores were predicted by cognitive flexibility scores while controlling for depression, anxiety, and stress. Planned exploratory Spearman’s rank-order correlations examined associations between performance on both cognitive flexibility tasks and self-reported cognitive–behavioral flexibility in everyday life (EDFLIX scores).

## Results

### Demographic and clinical characteristics

Participant demographics and clinical characteristics and mean scores for all variables are displayed in Table 1. WCST percentage perseverative errors in the sample were similar to those observed by van Autreve et al. [40] in comparable sample (age, *N*) of participants with AN. Scores for depression, anxiety, and stress were moderate, and scores for flexibility in everyday life were within the clinical range.

**Table 1:** Sample demographic characteristics and means (*M*) and standard deviations (*SD*) of all variables

Characteristic	*N*	*M* (*SD*) or %
Age (years)	45	29.6 (9)
Ethnicity		
Caucasian	35	78%
Asian	2	4%
Other	8	18%
Highest level of education		
University Postgraduate	10	22%
University Undergraduate	18	40%
Tertiary certificate	8	18%
Higher School Certificate (Grade 12)	9	20%
Diagnosis		
Current AN	22	49%
Past AN	23	51%
AN Diagnostic subtype		
AN-R	29	65%
AN-B/P	10	22%
OSFED/ Atypical AN	5	11%
Undisclosed	1	2%
Duration of illness (years)	44	9.8 (8.8)
Have received inpatient treatment	28	62%
Current BMI	41	21.7 (6)
WCST %PE	45	11.6 (4.9)
Switch cost	45	60.6 (57)
Memory span	45	20 (7.3)
Anti-saccade % errors	44	1.9 (2.1)
EDE-Q Global	45	2.3 (1.2)
DASS Depression	45	16 (12.3)
DASS Anxiety	45	11.4 (8.6)
DASS Stress	45	20.8 (9.7)
EDFLIX	45	117 (32)

*AN* anorexia nervosa, *AN-R* anorexia nervosa restricting type, *AN-B/P* anorexia nervosa binge–purge type, *BMI* Body Mass Index, *EDE-Q* Eating Disorder Examination Questionnaire, *EDFLIX* Eating Disorder Flexibility Index, *DASS* Depression Anxiety Stress Scale short form, *OSFED* Other Specified Eating and Feeding Disorder, WCST % PE = Wisconsin Card Sort Test percentage perseverative errors. Anti-saccade data for one participant was missing due to a technical issue. Providing BMI data was optional

### Regression analyses

Multiple linear regression tested whether switch costs predicted WCST scores while controlling for anti-saccade errors and memory span scores. The model indicated the three predictors explained 15% of the variance (*F*(3,43) = 2.42, *p* = 0.08), and switch costs were not a significant predictor of WCST perseverative error rate (*β* = − 0.03, *p* = 0.85). However, lower working memory span scores were a significant independent predictor of higher WCST perseverative error rates (*β* = − 0.32, *p* = 0.04).

Separate multiple linear regression models tested whether switch costs or WCST scores predicted EDE-Q global scores while controlling for depression, anxiety, and stress scores. The model including switch costs and mood variables was a significant predictor of EDE-Q global scores, explaining 29% of the variance (*F*(4,44) = 4.08, *p* < 0.01), but no variable was a significant independent predictor of higher EDE-Q global scores. The model including WCST perseverative error rates and mood variables was also a significant predictor of EDE-Q global scores, explaining 26% of the variance (*F*(4,44) = 3.48, *p* = 0.02), but higher stress scores were the only significant independent predictor of higher EDE-Q global scores (*β* = 0.42, *p* = 0.03). Full results of all multiple linear regression models are included in Supplementary Materials.

### Post-hoc analyses

To explore possible differences between participants with current (*n* = 22) versus past (*n* = 23) diagnoses of AN, we conducted a supplementary exploratory analysis directly comparing the two subgroups.

The two groups did not differ significantly in mean WCST scores: current (*M* = 11.96, *SD* = 4.58); Past (*M* = 11.24 *SD* = 5.27); *t* = 0.49,* p* = 0.31. Task switching costs were significantly higher for participants with a current diagnosis: current (*M* = 79.2, *SD* = 62.1), Past (*M* = 42.9, *SD* = 47.8);* t* = 2.20, *p* = 0.03. Including diagnostic status (current versus past) as a control predictor in the model regressing WCST perseverative errors on task switch costs and mood variables yielded the same pattern of significant results as the main analysis.

We also tested a regression model that excluded participants with partial threshold diagnoses (OSFED/AAN;* n* = 5). The pattern of significant results was identical to the main analysis.

#### Wisconsin Card Sort Test open-ended question results

Four participants reported they had done the task before, and 16 participants reported they found the task difficult. As expected, most of these participants reported difficulties establishing the sorting rule. More specifically, five participants reported difficulties identifying the possible dimensions; three appeared to have difficulty noticing the number dimension (“I found matching the number of shapes on the card the hardest to figure out”), one with the color dimension (“Took me a long time to work out that color could be a rule”), and one appeared to have general difficulty with the rules (“I didn't really understand what made it a match”). Four participants reported sensitivity to feedback (“when you don't like getting things wrong this is a challenge”; “it got quite frustrating when I made errors”; “tend to panic when getting things wrong”). One participant mentioned difficulties establishing the rule due to memory load (“hard to remember/focus what I was sorting based on”).

### Planned exploratory correlations

Neither of the cognitive flexibility measures or associated executive function measures were significantly associated with EDFLIX scores. Lower EDFLIX scores were strongly associated with higher EDE-Q global scores (*r* = − 0.78, *p* < 0.001), and moderately associated with higher stress scores (*r* = − 0.56, *p* < 0.01). Spearman’s correlations among all variables are included in Supplementary Materials.

## Discussion

The current study found no evidence that switch costs on a cued task-switching measure were associated with perseverative errors on the WCST in individuals with a lifetime diagnosis of AN, despite both being measures of cognitive flexibility. Contrary to our prediction, our results are not consistent with similar research in the general population [11, 25, 26], but are consistent with the results of van Autreve et al. [40] in participants with AN. The current data suggest these measures are not interchangeable measures of cognitive flexibility in participants with a lifetime diagnosis of AN. Results in this population appear to be specific to the task, rather than being generalizable to the construct of interest (i.e., *paradigm specificity*).

Because the WCST is a complex executive function test, we also assessed the relative contribution of inhibitory control and working memory capacity on performance. Working memory span scores were a significant independent predictor of WCST performance, suggesting that issues with updating working memory are a determinant of poorer performance on this task in participants with AN. Difficulties monitoring or updating working memory, which are needed to follow the sorting rule, could impact scores, and the current results are consistent with previous research which has failed to find typical differences between participants with AN and controls on the shorter 64-item WCST which reduces working memory load [38].

Participants’ self-reported experience of the WCST also appears to provide support for previous suggestions that perseverative errors could occur due to difficulties with other task-related processes, such as concept formation, sensitivity to feedback, and reversal learning [42]. Participants in the current study reported issues with establishing the sorting rule in the WCST which specifically related to identifying the potential sorting categories, and the negative effect of feedback. These results are consistent with research that found no differences in perseverative errors between participants with eating disorders and controls when explicit instructions and examples of the rule changes were provided [29], although these instructions are not standard for the administration of the WCST.

The results of the exploratory analysis suggest more systematic investigation of the associations between stress, clinical symptoms, and flexibility in AN is warranted. Lower self-reported flexibility was strongly associated with higher EDE-Q scores, and moderately associated with stress, consistent with past research in AN [6, 7]. Higher stress was also a significant independent predictor of higher EDE-Q scores. Meta-analytic results of general-population studies indicate that stress significantly impairs cognitive flexibility, and more severe stress is associated with more severe impairment [33].

Overall, the current results demonstrate that when controlling for mood variables, neither cognitive flexibility measure was a significant predictor of higher EDE-Q global scores. There was also no evidence of an association between either cognitive flexibility measure and self-reported cognitive–behavioral flexibility in everyday life, although this is consistent with the majority of comparisons between performance and self-reported flexibility in AN, and the general population [16, 23]. Together, these results do not provide adequate support for the assumption that poorer performance on cognitive flexibility tests is associated with the inflexible thoughts and behaviors observed in AN. Support for this assumption requires data which demonstrates clear associations between task performance and symptom severity [30].

For future cognitive flexibility research in eating disorders, this study has three key conclusions. First, common performance measures of cognitive flexibility may not be interchangeable in individuals with a lifetime diagnosis of AN, and future research should have a strong theoretical justification for the choice of measure. Second, cognitive flexibility is closely associated with other aspects of executive function, and these processes should be measured and controlled in the analysis, consistent with standard practice in the general population literature. Finally, this research highlights the importance of controlling for mood variables which are strongly associated with outcomes in individuals with eating disorders, and reiterates specific calls to control for mood in studies of cognitive flexibility in AN populations [1].

To reduce the influence of task-specific factors on the measurement of cognitive flexibility, large general population studies have adopted a latent-variable approach—testing across multiple methods and extracting a ‘general’ cognitive flexibility factor [25, 26]. Where sample size may not support this approach, such as in AN populations, reducing task-specific confounds and increasing the internal and test–retest reliability of measures can improve measurement. The current study suggests that task-switching may be a useful measure for future cognitive flexibility research in eating disorders to achieve these aims. Although all behavioral tasks are susceptible to the task impurity problem, the task-switching paradigm limits the issue in ways that may be of particular relevance to this population. Scores are not confounded by difficulty with reversal learning or low working memory capacity; rules are explained, the task is practiced, and is then cued or predictable based on location. There is no feedback, and thus no issue with feedback sensitivity, and any impact due to self-detected errors is reduced by the standard analysis procedure of removing error and post-error trial data before calculating switch costs. Task-switching paradigms also increase the reliability of measurement by collecting multiple observations across a large number of trials [4].

### Strength and limits

Strengths of the current study include preregistration of the analytic plan, concurrent measurement of multiple components of executive function and controlling for mood variables in the regression of clinical symptoms. Conclusions about the direction of effect cannot be made as this study was cross-sectional. Our sample included participants with a self-reported lifetime diagnosis of AN, including participants who reported a partial AN syndrome diagnosis, consistent with reports of poorer performance on cognitive flexibility tasks in participants with both current and past diagnoses [22] and across subtypes [34]. Inclusion of participants at differing stages of recovery resulted in a lower mean EDE-Q score than comparable in-patient studies of cognitive flexibility; however, the impact of these sample specifics is minimized in the current study due to the within-subjects design.

### What is already known on this subject?

Individuals with anorexia nervosa (AN) tend to perform more poorly on performance tests of cognitive flexibility than control groups, and prominent models include cognitive inflexibility as a key factor associated with the etiology and maintenance of the disorder. The most common measure of cognitive flexibility in AN is the WCST, but task-switching paradigms are beginning to be used in this population.

### What this study adds?

In a sample of individuals with a lifetime diagnosis of AN, there was no evidence of an association between cognitive flexibility measured as perseverative errors on the WCST and as switch costs in a cued task-switching paradigm. Results suggest greater perseverative errors may be partially determined by lower working memory capacity, and performance may be negatively impacted by sensitivity to feedback. After controlling for mood variables, neither cognitive flexibility measure was a significant predictor of symptom severity.

## Supplementary Information

Below is the link to the electronic supplementary material.

Supplementary file1 (DOCX 19 KB)

## Data Availability

Data are available on request from the first author subject to ethics approval.

## References

[1] Abbate-Daga G, Buzzichelli S, Marzola E, Aloi M, Amianto F, Fassino S (2015). Does depression matter in neuropsychological performances in anorexia nervosa? A descriptive review. Int J Eat Disord.

[2] Berner LA, Romero EM, Reilly EE, Lavender JM, Kaye WH, Wierenga CE (2019). Task-switching inefficiencies in currently ill, but not remitted anorexia nervosa. Int J Eat Disord.

[3] Broadway JM, Engle RW (2010). Validating running memory span: Measurement of working memory capacity and links with fluid intelligence. Behav Res Methods.

[4] Brysbaert M (2019). How many participants do we have to include in properly powered experiments? A tutorial of power analysis with reference tables. J Cogn.

[5] Dahlgren CL, Hage TW, Wonderlich JA, Stedal K (2019). General and eating disorder specific flexibility: development and validation of the eating disorder flexibility index (EDFLIX) questionnaire. Front Psycholdoi.

[6] Dann KM, Hay P, Touyz S (2022). Everyday flexibility and functional milestones in anorexia nervosa: survey results from a mixed community sample. Eat Weight Disord.

[7] Dann KM, Hay P, Touyz S (2022). Interactions between emotion regulation and everyday flexibility in anorexia nervosa: Preliminary evidence of associations with clinical outcomes. Eat Disord.

[8] Diamond A (2013). Executive Functions. Annu Rev Psychol.

[9] Fairburn CG, Beglin SJ (1994). Assessment of eating disorders: Interview or self-report questionnaire?. Int J Eat Disord.

[10] Faul F, Erdfelder E, Lang A-G, Buchner A (2007). G*Power 3: A flexible statistical power analysis program for the social, behavioral, and biomedical sciences. Behav Res Methods.

[11] Gamboz N, Borella E, Brandimonte MA (2009). The role of switching, inhibition and working memory in older adults' performance in the Wisconsin Card Sorting Test. Aging Neuropsychol Cogn.

[12] Grant DA, Berg E (1948). A behavioral analysis of degree of reinforcement and ease of shifting to new responses in a Weigl-type card-sorting problem. J Exp Psychol.

[13] Heaton RK, Chelune GJ, Talley JL, Kay GG, Curtiss G (1993). Wisconsin Card Sorting Test manual: revised and expanded.

[14] Henry JD, Crawford JR (2005). The short-form version of the Depression Anxiety Stress Scales (DASS-21): Construct validity and normative data in a large non-clinical sample. Br J Clin Psychol.

[15] Holliday J, Tchanturia K, Landau S, Collier D, Treasure J (2005). Is impaired set-shifting an endophenotype of anorexia nervosa?. Am J Psychiatry.

[16] Howlett CA, Wewege MA, Berryman C, Oldach A, Jennings E, Moore E, Karran EL, Szeto K, Pronk L, Miles S, Moseley GL (2021). Same room different windows? A systematic review and meta-analysis of the relationship between self-report and neuropsychological tests of cognitive flexibility in healthy adults. Clin Psychol Rev.

[17] King JA, Korb FM, Vettermann R, Ritschel F, Egner T, Ehrlich S (2019). Cognitive overcontrol as a trait marker in anorexia nervosa? Aberrant task- and response-set switching in remitted patients. J Abnorm Psychol.

[18] Lang K, Treasure J, Tchanturia K (2016). Is inefficient cognitive processing in anorexia nervosa a familial trait? A neuropsychological pilot study of mothers of offspring with a diagnosis of anorexia nervosa. The World Journal of Biological Psychiatry.

[19] Lovibond PF, Lovibond SH (1995). The structure of negative emotional states: comparison of the depression anxiety stress scales (DASS) with the beck depression and anxiety inventories. Behav Res Ther.

[20] Meiran N (1996). Reconfiguration of processing mode prior to task performance. J Exp Psychol Learn Mem Cogn.

[21] Meiran N, Hassin R, Ochsner K, Trope Y (2010). Task switching: mechanisms underlying rigid vs. flexible self-control. Self control in society, mind, and brain.

[22] Miles S, Gnatt I, Phillipou A, Nedeljkovic M (2020). Cognitive flexibility in acute anorexia nervosa and after recovery: a systematic review. Clin Psychol Rev.

[23] Miles S, Nedeljkovic M, Sumner P, Phillipou A (2022). Understanding self-report and neurocognitive assessments of cognitive flexibility in people with and without lifetime anorexia nervosa. Cogn Neuropsychiatry.

[24] Millisecond Software (2021) [Computer software]. https://www.millisecond.com. Accessed Jan 2021

[25] Miyake A, Emerson MJ, Friedman NP (2000) Assessment of executive functions in clinical settings: problems and recommendations. Paper presented at the Seminars in speech and language. 10.1055/s-2000-7563 10879548

[26] Miyake A, Friedman NP, Emerson MJ, Witzki AH, Howerter A, Wager TD (2000). The unity and diversity of executive functions and their contributions to complex “Frontal lobe” tasks: a latent variable analysis. Cogn Psychol.

[27] Mond JM, Hay PJ, Rodgers B, Owen C, Beumont PJV (2004). Validity of the Eating Disorder Examination Questionnaire (EDE-Q) in screening for eating disorders in community samples. Behav Res Ther.

[28] Monsell S (2003). Task switching. Trends Cogn Sci.

[29] Pignatti R, Bernasconi V (2013). Personality, clinical features, and test instructions can affect executive functions in eating disorders. Eat Behav.

[30] Ravizza SM, Salo RE, Grange J, Houghton G (2014). Task switching in psychiatric disorders. Task switching and cognitive control.

[31] Schmidt U, Treasure J (2006). Anorexia nervosa: valued and visible. A cognitive-interpersonal maintenance model and its implications for research and practice. Br J Clin Psychol.

[32] Sereno AB, Holzman PS (1995). Antisaccades and smooth pursuit eye movements in schizophrenia. Biol Psychiat.

[33] Shields GS, Sazma MA, Yonelinas AP (2016). The effects of acute stress on core executive functions: A meta-analysis and comparison with cortisol. Neurosci Biobehav Rev.

[34] Smith KE, Mason TB, Johnson JS, Lavender JM, Wonderlich SA (2018). A systematic review of reviews of neurocognitive functioning in eating disorders: the state-of-the-literature and future directions. Int J Eat Disord.

[35] Stedal K, Broomfield C, Hay P, Touyz S, Scherer R (2021). Neuropsychological functioning in adult anorexia nervosa: A meta-analysis. Neurosci Biobehav Rev.

[36] Stoet G (2010). PsyToolkit: a software package for programming psychological experiments using Linux. Behav Res Methods.

[37] Stoet G (2016). PsyToolkit: a novel web-based method for running online questionnaires and reaction-time experiments. Teach Psychol.

[38] Talbot A, Hay P, Touyz S (2015). Exploring the relationship between cognitive style and daily functioning in patients with anorexia nervosa. Advances in Eating Disorders.

[39] Treasure J, Schmidt U (2013). The cognitive-interpersonal maintenance model of anorexia nervosa revisited: a summary of the evidence for cognitive, socio-emotional and interpersonal predisposing and perpetuating factors. J Eat Disord.

[40] van Autreve S, de Baene W, Baeken C, van Heeringen C, Vervaet M (2013). Do restrictive and bingeing/purging subtypes of anorexia nervosa differ on central coherence and set shifting?. Eur Eat Disord Rev.

[41] Van Autreve S, De Baene W, Baeken C, van Heeringen K, Vancayseele N, Vervaet M (2016). Differential neural correlates of set-shifting in the bingeing-purging and restrictive subtypes of anorexia nervosa: an fMRI study. Eur Eat Disord Rev.

[42] Wildes JE, Forbes EE, Marcus MD (2014). Advancing research on cognitive flexibility in eating disorders: the importance of distinguishing attentional set-shifting and reversal learning. Int J Eat Disord.

[43] Wu M, Brockmeyer T, Hartmann M, Skunde M, Herzog W, Friederich HC (2014). Set-shifting ability across the spectrum of eating disorders and in overweight and obesity: a systematic review and meta-analysis. Psychol Med.

