# Supplementary material for: Measuring cognitive flexibility in anorexia nervosa: Wisconsin Card Sorting Test versus cued task-switching
Source: Eat Weight Disord. 2023 Jul 18;28(1):60. doi: 10.1007/s40519-023-01589-6 (PMC10354129; doi:10.1007/s40519-023-01589-6)
Supplement: Supplementary file 1 — Supplementary file1 (DOCX 19 KB) [file 40519_2023_1589_MOESM1_ESM.docx]

**Supplementary Materials**

Table S1. Spearman’s rank order correlations, means (*M*) and standard deviations (*SD*) of all variables.

| **Variable** | ***n*** | | ***M*** | ***SD*** | **1** | **2** | **3** | **4** | **5** | **6** | **7** | **8** |
| --- | --- | --- | --- | --- | --- | --- | --- | --- | --- | --- | --- | --- |
| **1. WCST % PE** | | 45 | 11.6 | 4.9 | - |  |  |  |  |  |  |  |
| **2. Switch cost** | | 45 | 60.6 | 57.6 | -.15 | - |  |  |  |  |  |  |
| **3. Memory Span** | | 45 | 20 | 7.3 | -.22 | .07 | - |  |  |  |  |  |
| **4. Anti-saccade errors (%)** | | 44 | 1.9 | 2.1 | .234 | -.21 | -.01 | - |  |  |  |  |
| **5. EDE-Q Global** | | 45 | 2.3 | 1.2 | -.10 | **.32*** | .18 | <.01 | - |  |  |  |
| **6. EDFLIX** | | 45 | 117.2 | 32.6 | .15 | -.24 | -.15 | .03 | **-.78**** | - |  |  |
| **7. DASS Depression** | | 45 | 16 | 12.3 | -.02 | -.01 | -.08 | .04 | **.36*** | **-.42**** | - |  |
| **8. DASS Anxiety** | | 45 | 11.4 | 8.6 | -.03 | -.02 | <.01 | -.02 | .20 | -.24 | **.32*** | - |
| **9. DASS Stress** | | 45 | 20.8 | 9.7 | -.12 | .25 | .14 | -.03 | .**42**** | **-.56**** | **.31*** | **.64**** |

Note: Significant *p*-values are highlighted in bold; ** Correlation is significant at the .01 level (2-tailed) * Correlation is significant at the .05 level (2-tailed). Abbreviations: WCST % PE = Wisconsin Card Sort Test percentage perseverative errors, EDE-Q = Eating Disorder Examination Questionnaire, EDFLIX = Eating Disorder Flexibility Index, DASS = Depression Anxiety Stress Scale short form.

Table S2. Regression analyses.

| **WCST % PE** | | | | | |
| --- | --- | --- | --- | --- | --- |
|  | *b* | SE*_b_* | *β* | *t* | *p* |
| Intercept | 11.5 | .71 |  | 16.2 | <.001 |
| Switch cost | <.01 | .01 | -.03 | -.19 | .85 |
| Memory | **-.21** | **.10** | **-.32** | **-2.2** | **.04** |
| Anti-saccade | .46 | .35 | .20 | 1.3 | .19 |
|  |  |  |  |  |  |
|  | *R*^2^ = .15, *F* (3,43) = 2.4, *p* = .08 | | | | |

| **EDE-Q Global** | | | | | | **EDE-Q Global** | | | | | | |
| --- | --- | --- | --- | --- | --- | --- | --- | --- | --- | --- | --- | --- |
|  | *b* | SE*_b_* | *β* | *t* | *p* |  | *b* | SE*_b_* | *β* | *t* | *p* |  |
| Intercept | 2.3 | .16 |  | 14.3 | <.001 | Intercept | 2.6 | .30 |  | 8.4 | <.001 |  |
| Switch cost | .01 | <.01 | .23 | 1.6 | .12 | WCST % PE | -.02 | .02 | -.12 | -.83 | .41 |  |
| Depression | .03 | .02 | .27 | 1.8 | .08 | Depression | .03 | .02 | .27 | 1.7 | .09 |  |
| Anxiety | -.01 | .03 | -.09 | -.46 | .65 | Anxiety | -.03 | .03 | -.18 | -.94 | .35 |  |
| Stress | .04 | .03 | .34 | 1.8 | .09 | Stress | **.05** | **.02** | **.42** | **2.2** | **.03** |  |
|  | ***R*^2^ = .29, *F* (4,44) = 4.1, *p* = <.01** | | | | |  | ***R*^2^ = .26, *F* (4,44) = 3.5, *p* = .02** | | | | |  |

Note: *b* = unstandardized regression coefficient; *β* = standardized regression coefficient. All predictor variables are mean centered. Abbreviations: EDE-Q = Eating Disorder Examination Questionnaire, WCST % PE = Wisconsin Card Sort Task percentage perseverative errors.
